# Synergistic Effects of Targeted PI3K Signaling Inhibition and Chemotherapy in Liposarcoma

**DOI:** 10.1371/journal.pone.0093996

**Published:** 2014-04-02

**Authors:** Shang Guo, Hector Lopez-Marquez, Kenneth C. Fan, Edwin Choy, Gregory Cote, David Harmon, G. Petur Nielsen, Cao Yang, Changqing Zhang, Henry Mankin, Francis J. Hornicek, Darrell R. Borger, Zhenfeng Duan

**Affiliations:** 1 Center for Sarcoma and Connective Tissue Oncology, Massachusetts General Hospital, Boston, Massachusetts, United States of America; 2 Shanghai Sixth People’s Hospital affiliated to Shanghai Jiao Tong University, Shanghai, China; 3 Translational Research Laboratory, Massachusetts General Hospital, Boston, Massachusetts, United States of America; 4 Department of Pathology, Massachusetts General Hospital, Boston, Massachusetts, United States of America; 5 Department of Orthopaedics, Union Hospital, Huazhong University of Science and Technology, Wuhan, China; H.Lee Moffitt Cancer Center & Research Institute, United States of America

## Abstract

While liposarcoma is the second most common soft tissue malignant tumor, the molecular pathogenesis in this malignancy is poorly understood. Our goal was therefore to expand the understanding of molecular mechanisms that drive liposarcoma and identify therapeutically-susceptible genetic alterations. We studied a cohort of high-grade liposarcomas and benign lipomas across multiple disease sites, as well as two liposarcoma cell lines, using multiplexed mutational analysis. Nucleic acids extracted from diagnostic patient tissue were simultaneously interrogated for 150 common mutations across 15 essential cancer genes using a clinically-validated platform for cancer genotyping. Western blot analysis was implemented to detect activation of downstream pathways. Liposarcoma cell lines were used to determine the effects of PI3K targeted drug treatment with or without chemotherapy. We identified mutations in the *PIK3CA* gene in 4 of 18 human liposarcoma patients (22%). No *PIK3CA* mutations were identified in benign lipomas. Western blot analysis confirmed downstream activation of AKT in both *PIK3CA* mutant and non-mutant liposarcoma samples. PI-103, a dual PI3K/mTOR inhibitor, effectively inhibited the activation of the PI3K/AKT in liposarcoma cell lines and induced apoptosis. Importantly, combination with PI-103 treatment strongly synergized the growth-inhibitory effects of the chemotherapy drugs doxorubicin and cisplatin in liposarcoma cells. Taken together, these findings suggest that activation of the PI3K/AKT pathway is an important cancer mechanism in liposarcoma. Targeting the PI3K/AKT/pathway with small molecule inhibitors in combination with chemotherapy could be exploited as a novel strategy in the treatment of liposarcoma.

## Introduction

Liposarcoma is the second most common soft tissue malignant tumor, accounting for 15% of all adult soft tissue sarcomas. The male to female age ratio at the time of diagnosis is 57 to 43 years, with the mean age being 51±17 years (range 5 to 94 years) [1]–[4]. Liposarcoma usually arises in lipoblast cells in a deep soft tissue, such as those inside the thigh or in the retroperitoneum. Patient survival of liposarcoma is related to the degree of malignancy of the cell types, localization, and size of the primary tumor and treatment protocols [2]. According to the World Health Organization and others, liposarcoma is currently subclassified into four groups: well differentiated, dedifferentiated, myxoid/round cell, and pleomorphic [4], [5]. In contrast, lipoma is a benign tumor which is composed of adipose tissue and is the most common form of soft tissue tumor [6], [7]. Although some genetic studies have provided insight into the mechanism of liposarcoma and lipoma development, the exact pathogenesis remains largely unknown [8]–[10]. Oncogenesis is a multistep process largely involving the activation of oncogenes and/or the inactivation of tumor suppressor genes. However, this process has rarely been investigated in liposarcoma relative to lipoma. Interestingly, previous studies, molecular abnormalities associated with liposarcoma have been reported, such as amplification of the *mdm2* gene and overexpression of the mdm2 protein emerging as the most frequent abnormality in dedifferentiated liposarcoma [11]–[13]. Although *HRAS* mutations have been reported in malignant fibrous histiocytoma (MFH), leiomyosarcoma, and rhabdomyosarcoma, *HRAS* mutation is a relatively uncommon event in liposarcoma [14]. Furthermore, liposarcomas showed neither *TP53* gene mutations nor altered *MYC* gene expression [15]. These results indicate that the *HRAS* and *MYC* oncogenes and the *TP53* tumor suppressor gene may not play a major role in the etiology of liposarcomas. In contrast, zebrafish expressing constitutively active AKT2 in mesenchymal progenitors develop well-differentiated liposarcoma that closely resembles the human disease [16]. Recent study has shown activating PIK3CA mutations were found in 14% of liposarcoma [17]. In a novel established dedifferentiated liposarcoma xenograft mouse model, PTEN down-regulation has been shown as a malignant signature and response to PI3K pathway inhibition [18]. These studies suggest further identification of critical carcinogenic driver mechanisms in liposarcoma tumor specimens may predict patient outcomes and provide potential targets for therapeutic intervention.

For the treatment of liposarcoma, surgical resection remains the main modality for curative therapy. However, large liposarcomas in the extremity or retroperitoneal are associated with high local recurrence (15% and 75%) and poor overall survival [1], [3]. Incorporation of neo-adjuvant approaches such as chemotherapy or radiotherapy may improve local control; however, though little progress has been made on improving the survival in this disease in the past 20 years (1, 16). The aim of this study is to identify unique genetic variations in liposarcoma by using a clinically-validated SNaPshot cancer genotyping platform that evaluates150 common hotspot mutations across 15 cancer driver genes [19], [20], ultimately with the overall goal of finding new therapeutically-relevant targets for liposarcoma patients.

## Materials and Methods

### Ethic Statement and Liposarcoma Tumor Samples

Fresh-frozen liposarcoma and lipoma patient specimens were obtained from the clinical archives of the Sarcoma Tumor Bank of Massachusetts General Hospital. A cohort of 18 high-grade liposarcomas and 19 benign lipomas obtained across multiple disease sites were selected for this study. Patient medical record information was also collected for this study. Institutional review board approval was obtained from the Partners Human Research Office (2007P-002464). Patient records/information was anonymized and de-identified prior to analysis.

### Cell Lines and Cell Culture

The human liposarcoma cell lines SW872 (an undifferentiated liposarcoma, ATCC catalog number: HTB-92) and SW982 (another undifferentiated liposarcoma as evaluated by histopathology, ATCC catalog number: HTB-93) were purchased from the ATCC (Rockville, MD). These cell lines were cultured in RPMI 1640 (Invitrogen, Carlsbad, CA) supplemented with 10% FBS, 100 units/ml penicillin and 100 μg/ml streptomycin (Invitrogen). Cells were incubated at 37°C in a 5% CO_2_-95% air atmosphere and passaged when near confluent monolayers were achieved using Trypsin-EDTA solution. Cells were free of Mycoplasma contamination, as tested by the MycoAlert Mycoplasma Detection Kit from Cambrex (Rockland, ME).

### Extraction of Genomic DNA

Extraction of DNA from liposarcoma tumor tissues and cell lines was performed using the QIAamp DNA Micro kit (Qiagen, Valencia, CA). The extraction was carried out according to the manufacturer’s instructions. Briefly, liposarcoma tumor tissue samples (approximately 50 mg) or cell pellets from cultured cell lines (approximately 8 mg) were transferred to a 1.5 mL microcentrifuge tube and 180 μL of buffer ATL (Qiagen) was immediately added. After equilibrating to room temperature (25°C), 20 μL of proteinase K was added and mixed by vortexing for 15 seconds. The sample tube was incubated at 56°C overnight until the sample was completely lysed. The next day, 200 μL buffer AL (Qiagen) was added and mixed by vortexing for 15 seconds. Subsequently, 200 μL of ethanol (96%–100%) was added. The mixture obtained was loaded on a QIAampMiniElute spin column and washed with AW1 buffer, followed by AW2 buffer (Qiagen). DNA was eluted with 60 μL of bufferAE (Qiagen) and preserved at −20°C until use. Quantity and quality of the DNA was determined using a spectrophotometer (DU640, Beckman Instruments, Inc, Fullerton, CA) and agarose gel electrophoresis.

### Liposarcoma Genetic Mutation Analysis

A clinically-validated cancer genotyping platform that evaluates150 common hotspot mutations across 15 cancer driver genes (*AKT1*, *APC*, *BRAF*, *CTNNB1*, *EGFR*, *ERBB2*, *IDH1*, *KIT*, *KRAS*, *MAP2K1*, *NOTCH1*, *NRAS*, *PIK3CA*, *PTEN*, *TP53*) was employed, using DNA isolated from frozen human liposarcoma and lipoma samples. The assay is based on the SNaPshot multiplex platform (Applied biosystems, Carlsbad, CA), as described previously [19], [20]. Sequence analysis of mutational hotspots mapping within the PIK3CA adaptor–binding, helical and kinase domains was conducted by direct sequencing of M13-tagged PCR products generated using the following primer pairs: PIK3CA exon 1, 5′ tgtaaaacgacggccagtTGCTTTGGGACAACCATACA- 3′ (forward) and 5′-caggaaacagctatgaccttttagaaagggacaacagttaagc-3′ (reverse); exon 9, 5′-TGTAAAACGACGGCCAGTctgtgaatccagaggggaaa-3′ (forward) and 5′-CAGGAAACAGCTATGACCcatgctgagatcagccaaat-3′ (reverse); exon 20, 5′-tgtaaaacgacggccagtcatttgctccaaactgacca-3′ (forward) and 5′ caggaaacagctatgaccTGTGGAATCCAGAGTGAGCTT-3′ (reverse). Thermocycling was conducted at 95°C for 8 minutes, followed by 45 cycles of 95°C for 20 seconds, annealing at 58°C (exon 1 and 20) or at 68°C (exon 9) for 30 seconds and 72°C for 1 minute, and one last cycle of 72°C for 3 minutes. Sequencing was conducted using the incorporated M13 primer tag, as previously described [19].

### Western Blot Analysis of Phosphoinositide-3-kinase (PI3K) Pathway Proteins

The human AKT, phosphorylated AKT (pAKT) (threonine [Thr]308), PI3K p110α, phosphorylated 4E binding protein 1 (p4EBP1) (Thr37/46), BcL-XL, Cytochrome c, caspase 3, mTOR, phosphorylated mTOR (pmTOR) antibodies were purchased from Cell Signaling Technologies (Dedham, MA). The mouse monoclonal antibody to human actin was purchased from Sigma-Aldrich (St. Louis, MO). Western blot analysis was performed as previously described, with modifications [21]. Briefly, cells were lysed in1X radioimmunoprecipitation assay (RIPA) lysis buffer (Upstate Biotechnology, Lake Placid, NY) and protein concentration was determined by the DC Protein Assay (Bio-Rad, Hercules, CA). Total protein (25 μg) was resolved on NuPage 4% to 12% Bis-Tris gels (Invitrogen, Carlsbad, CA). After electrophoresis, proteins were transferred to PROTRAN nitrocellulose transfer membranes (Whatman GmbH, Germany). Membranes were blocked for 2 hours at 4°C with Odyssey Blocking Buffer (LI-COR Biosciences, Lincoln, NE), then incubated at 4°C overnight with primary antibodies diluted in Odyssey Blocking Buffer. After incubating with primary antibodies, membranes were washed with TBS-T (containing 0.1% Tween 20) three times, 5 minutes per each. Membranes were then incubated at room temperature with rocking for 1 hour with IRDye800CW-conjugated goat anti-rabbit IgG and IRDye680-conjugated goat anti-mouse IgG secondary antibodies (LI-COR Biosciences) diluted in Odyssey Blocking Buffer. Blots were then washed three times with TBS-T and rinsed with PBS. The levels of expressed proteins were visualized by scanning the membrane on an Odyssey Infrared Imaging System (LI-COR Biosciences) with both 700- and 800-nm channels.

**Table 1:** Characteristics of Patients with Liposarcoma and Lipoma (*n* = 37).

Characteristics	Numbers	Percentage	PIK3CA Mutation
**Age at diagnosis, years**			
Median	51	N/A	
Range	20–74	N/A	
**Sex**			
Male	21	57	
Female	16	43	
**Liposarcoma**	18	49	
Well-differentiated	2	11	0
Myxoid	10	56	4
Pleiomorphic	2	11	0
Dedifferentiated	2	11	0
Mixed	2	11	0
**Lipoma**	19	51	0
**Recurrence**	2	11	
**Metastasis**	3	17	

### Apoptosis Assay

The dual PI3K/AKT and mTOR inhibitor PI-103 (3-(4-(4-Morpholinyl)pyrido[3′,2′∶4,5]furo[3,2-d]pyrimidin-2-yl]phenol) was purchased from EMD chemicals, Inc (Gibbstown, NJ) and dissolved in DMSO. Liposarcoma lines SW872 and SW982 were seeded at 8000 cells/per well in a 96-well plate for 24 hours before treatment with different concentrations of PI-103 for 48 hours. The cells were then lysed by adding 10 μl of 10% NP-40 per well. Apoptosis was evaluated by a caspase-cleaved keratin 18**-**based quantification kit (M30-Apoptosense ELISA assay), as per manufacturer’s instructions (Peviva AB, Bromma, Sweden). This assay detects the 21-kDa fragment of cytokeratin 18 that is only revealed after caspase cleavage of the protein. M30-Apoptosense ELISA kit has been used for both epithelial (ovarian cancer) and non epithelial (osteosarcoma) cells apoptosis assays [22], [23]. Apoptosis was also evaluated by Western blot using whole-cell lysates immunoblotted with specific antibodies to PARP (Cell Signaling Technologies, Cambridge, MA) and its cleavage products.

### MTT (3-(4,5-Dimethylthiazol-2-yl)-2,5- diphenyltetrazolium Bromide) Cytotoxicity Assay

PI-103 and chemotherapy drug cytotoxicity was assessed *in vitro* using the MTT (3-(4,5-dimethylthiazol-2-yl)-2,5-diphenyltetrazolium bromide) assay. Briefly, 2×10^3^ cells per well were plated in 96-well plates in culture medium (RPMI 1640 supplemented with 10% fetal bovine serum and penicillin/streptomycin) and treated with 0.5 μM PI-103, 0.5 μM doxorubicin, 1 μM cisplatin or a combination of either of the cytotoxics and PI-103. After 4 days of culture, 10 μl of MTT (Sigma-Aldrich, 5 mg/ml in PBS) was added to each well and the plates were incubated for 4 h. The resulting formazan product was dissolved with HCL-isopropanol and the absorbance at a wavelength of 490 nm (A490) was read on a SPECTRAmaxR Microplate Spectrophotometer (Molecular Devices).

### Statistical Analysis

GraphPad PRISM1 4 software from GraphPad Software, Inc. was used to statistically analyze the data. Errors were SD of averaged results and p values <0.05 were accepted as a significant difference between means.

## Results

### Liposarcoma Patients and Samples

We identified 18 cases of liposarcoma and 19 cases of lipoma with sufficient tissues for molecular analysis from the clinical archives of the Sarcoma Tumor Bank at the Massachusetts General Hospital (Table. 1). The most common type of liposarcoma in this cohort was myxoid, at a frequency of 56% (n = 10). There were also 2 samples each for the well-differentiated, pleiomorphic, dedifferentiated and mixed liposarcoma subtypes (Table. 1). The median patient age at disease appearance was 51 years (range, 20 to 74 years) and 21 (57%) patients were male and 16 (43%) were female.

### Identification of PIK3CA Mutation in Liposarcoma

**Figure 1:**
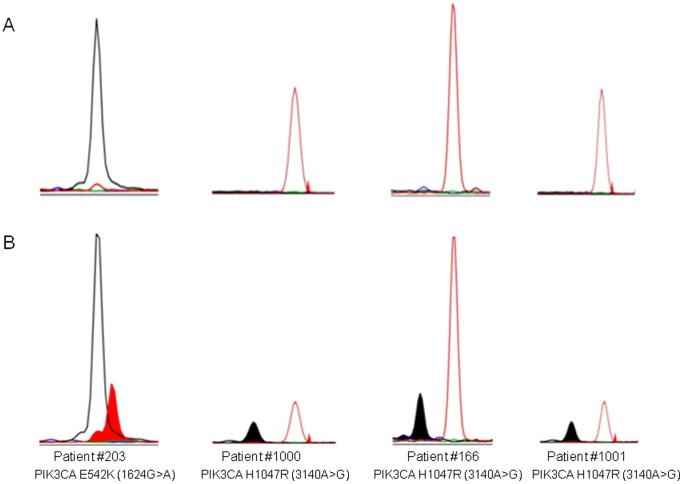
Mutational profiling of liposarcoma in four patients using a clinical mutational profiling assay. DNA was extracted from human liposarcoma and lipoma specimens and were evaluated for somatic mutations across 15 essential cancer genes (150 point mutations). The four sections on the bottom (B) are portions of the electropherogram that identify the *PIK3CA* gene mutations identified across 4 patient liposarcoma samples. For reference, each section is accompanied by genotyping data obtained from a non-cancerous control sample directly above each patient’s plot (A).

Mutational profiling was performed on the liposarcoma and lipoma patient samples, querying for 150 known somatic driver mutations. A BRAF V600E (1799T>A) and PTEN deletion was found in the liposarcoma cell line SW872, as previously described (http://www.sanger.ac.uk/perl/genetics/CGP/cosmic?action=sample&name=SW872). No mutations were found in any of the patient lipoma samples. Conversely, activating mutations in the *PIK3CA* gene (PI3-kinase, p110 alpha subunit) were observed in four cases (22%) of liposarcoma (Figure 1) One tumor sample (Patient #203, female) harbored the E542K (1624G>A) mutation located with the helical domain of PIK3CA, and three cases (Patient #166, 1000, 1001, all male) carried the H1047R (3140A>G) mutation located with the PIK3CA kinase domain. All four PIK3CA mutations were identified in myxoid liposarcoma out of 10 total samples with this histological subtype (33%).

### Activation of the PI3K Pathway in Liposarcoma Tumor Tissues

**Figure 2:**
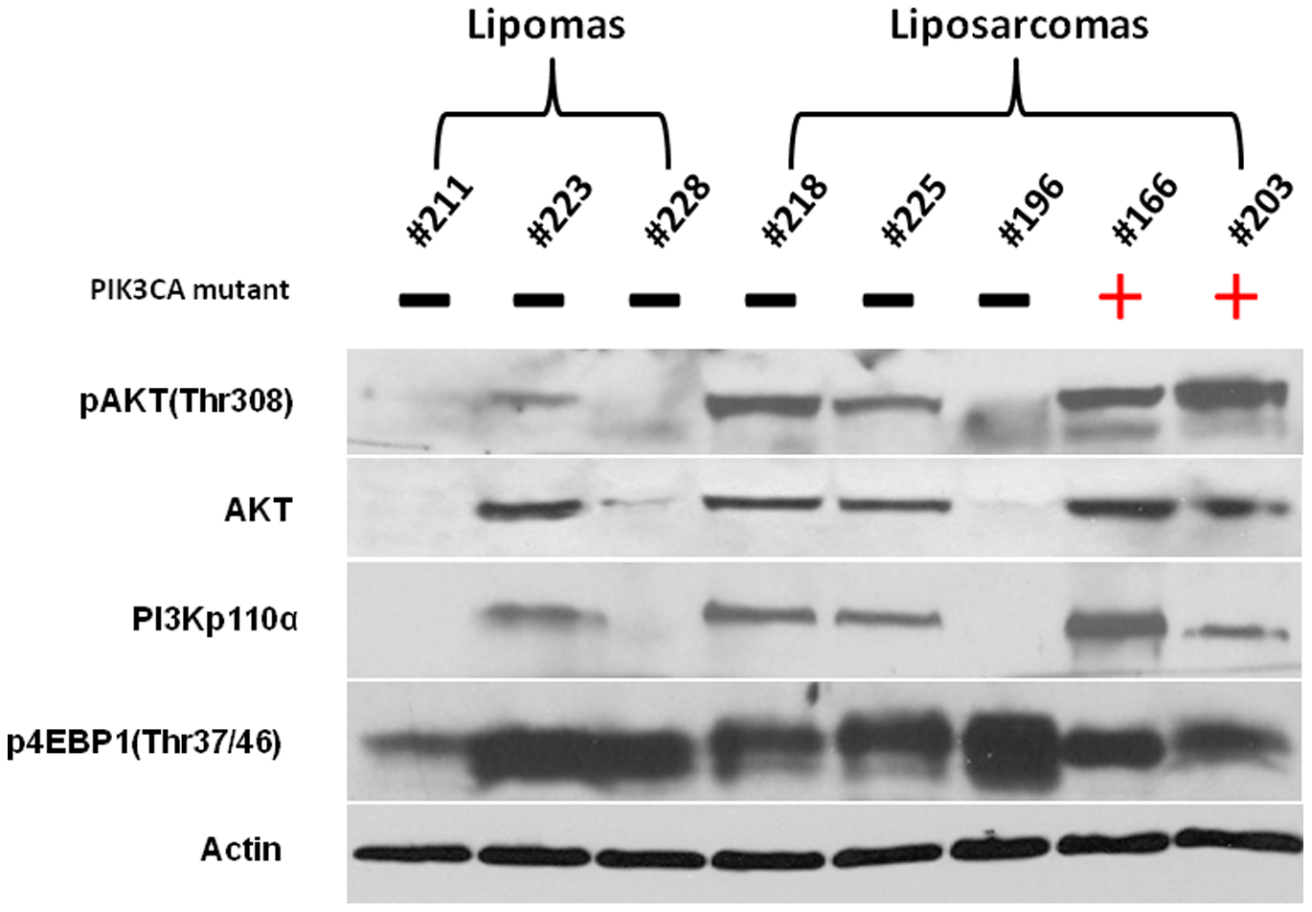
Western blot analysis of PI3K pathway protein expression levels of PI3K p110α, AKT, pAKT (Thr308), p4EBP1 (Thr37/46) in liposarcoma and lipoma tissues. For Western blot analysis, 25 μg of cellular proteins was subjected to immunoblotting with specific antibodies as described in Materials and Methods.

The human PI3K proteins are heterodimeric molecules composed of a regulatory and a catalytic subunit. *PIK3CA* is the gene coding for the p110α protein of PI3K that is a class I PI3K catalytic subunit [24]. AKT is a major downstream effector of PI3K and AKT/mTOR activation and can regulate a number of cellular processes that drive the tumorigenic process, such as inhibition of apoptosis, expression of survival genes, cell cycle progression, protein translation, and cell growth and proliferation [17], [24], [25]. Western blot analysis was used to determine the relative extent of PI3K pathway activation in 2 liposarcoma cases that carried an activating *PIK3CA* mutation compared with 3 liposarcoma samples for which *PIK3CA* mutations were not observed, as well as 3 lipoma samples (Figure 2). Because it is known that PI3K activates both the AKT and mTOR pathways, the relative levels of pAKT protein was used to gauge AKT activity and levels of p4EBP1 was used as an indicator mTOR activity. Using this strategy, only 1 out of 3 lipoma samples demonstrated pathway activity as evidenced by elevated levels of pAKT and p4EBP1. Conversely, 2 out of 2 *PIK3CA*-mutant and 2 out of 3 *PIK3CA*-wild type liposarcoma samples demonstrated high levels of pAKT. Furthermore, all 5 liposarcoma samples revealed highly expressed p4EBP1, regardless of *PIK3CA* mutational status. These data suggest that activation of AKT/mTOR signaling is likely an essential tumorigenic driver, where *PIK3CA* gene mutation may serve as one of several mechanism of pathway activation.

### Inhibition of PI3K Pathway Proteins by PI-103 in Liposarcoma Cell Lines

**Figure 3:**
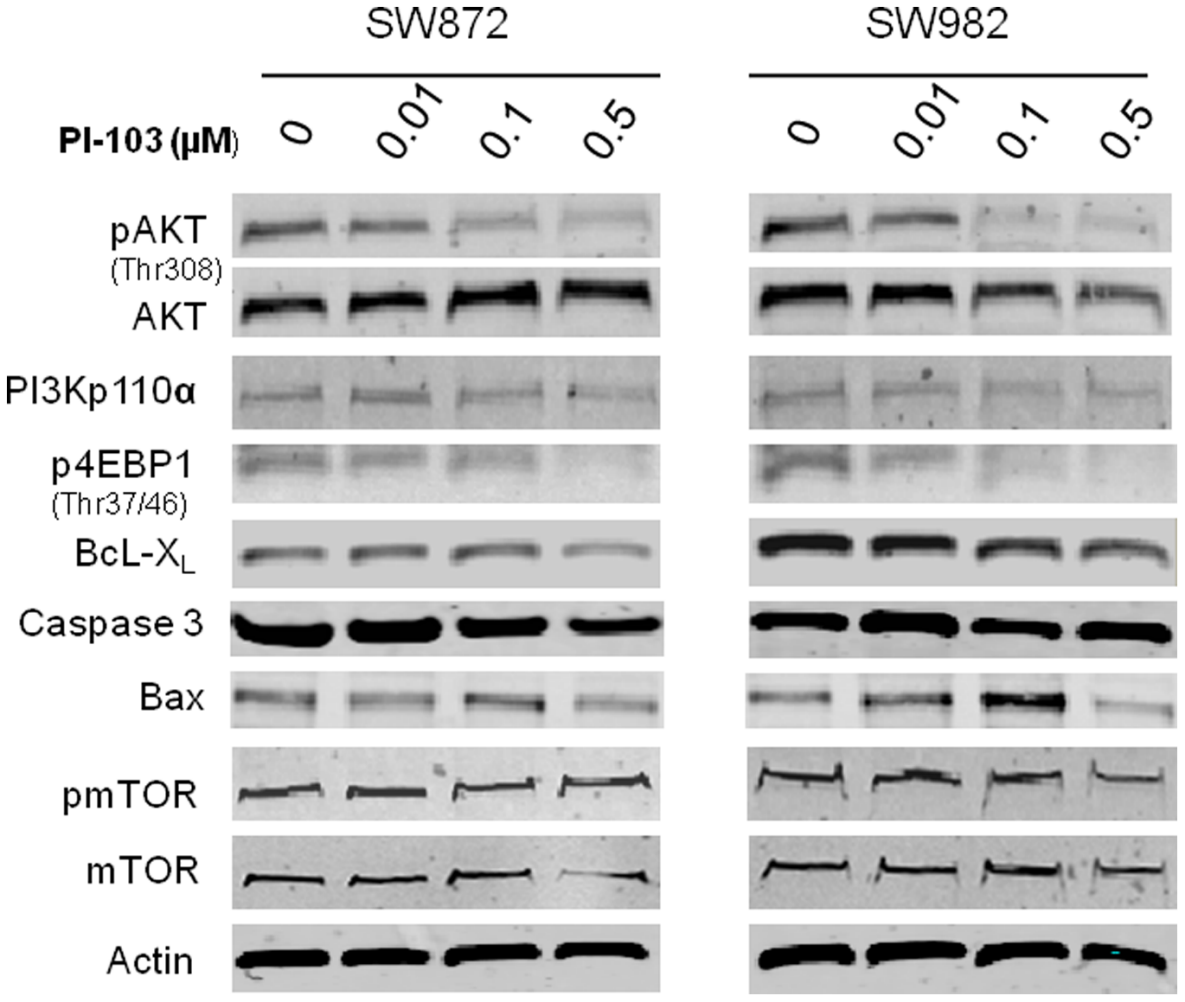
Effects of PI-103 on PI3K/mTOR in liposarcoma cell lines. SW872 and SW982 cells were treated with different concentrations of PI-103 for 24 hours. The effects of PI-103 on PI3K/mTOR pathway protein expression levels were determined by West blot analysis. Note the significant decrease in pAKT and p4EBP1 expression in both of liposarcoma cell lines post PI-103 treatment.

Although the two liposarcoma cell lines SW872 and SW982 did not show *PIK3CA* mutation by mutational profiling, both cell lines exhibited high baseline levels of PI3K signaling **(** Figure 3 **)**. Western blot analysis demonstrated that exposure to PI-103, a dual inhibitor of AKT and mTOR [26], [27], reduced AKT phosphorylation (pAKT) and 4EBP1 phosphorylation (p4EBP1) in a dose-dependent manner in both cell lines (Figure 3). In addition, expression levels of the anti-apoptosis protein BcL-X_L_ was also reduced by PI-103. pmTOR was not inhibited by PI-103 at the doses given.

### Induction of Apoptosis by PI-103 in Liposarcoma Cell Lines

**Figure 4:**
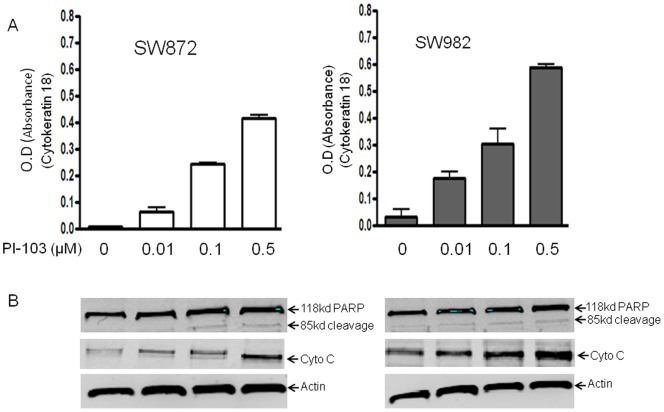
PI3K/mTOR inhibitor PI-103 induces apoptosis in liposarcoma cells. SW872 and SW982 cells were treated with different concentration of PI-103 for 48 hours. The apoptotic effects of PI-103 on liposarcoma cells were determined by M-30-Apoptosense ELISA kit and Western blot analysis of PARP cleavage. (A) M30-Apoptosense ELISA assay was done as described in Materials and Methods. (B) Total cellular proteins were subjected to immunoblotting with specific antibodies to PARP and β-actin as described in Materials and Methods.

The effect of PI-103 on the induction of apoptosis in liposarcoma cell lines SW872 and SW982 was assessed by M-30-Apoptosense ELISA and by evaluating PARP cleavage. PARP cleavage was detected 24 hours following incubation with PI-103 (Figure 4A). A dose-response analysis revealed the appearance of PARP cleavage products in the presence of 0.1 μmol of PI-103 when the cells were allowed to incubate for 24 hours Treatment of liposarcoma cells with PI-103 also showed moderate effect on cytochrome c release (Figure 4B).

### Synergistic Effect of PI-103 on Cisplatin and Doxorubicin Sensitivity in Liposarcoma Cell Lines

**Figure 5:**
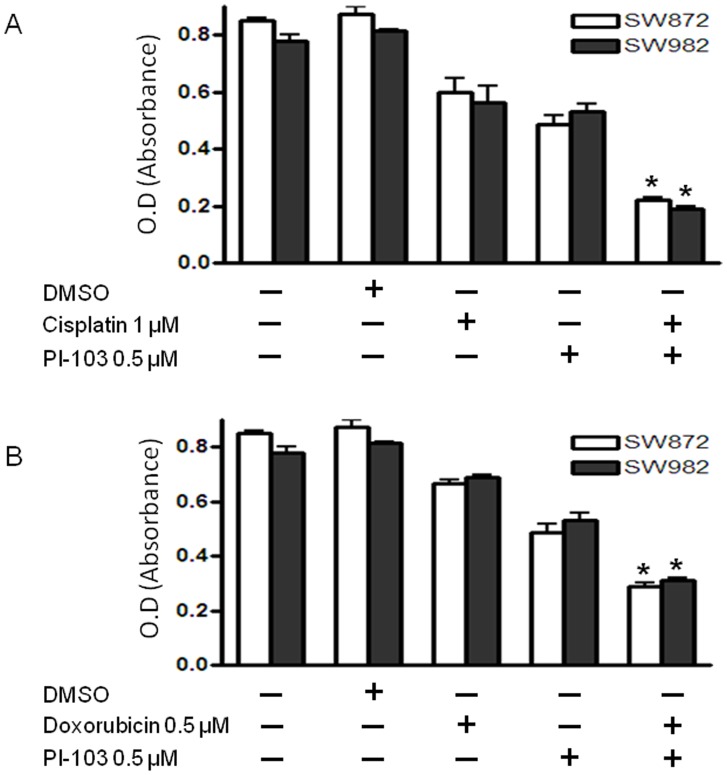
Synergistic effects of PI3K/mTOR inhibitor PI-103 on chemotherapy drug in liposarcoma cell lines. (A) SW872 or SW982 cells was exposed to combinations of cisplatin/PI-103. The combination caused a synergistic increase in the cell death. (B) SW872 or SW982 cells was exposed to combinations of doxorubicin/PI-103. The combination also caused a synergistic increase in the cell death.

Constitutive activation of PI3K/mTOR signaling may contribute to the survival advantage of human cancer cells, in part through the induction of antiapoptotic regulatory proteins. We hypothesized that inhibition of AKT/mTOR pathway in liposarcoma would lower the apoptotic threshold and increase chemotherapy sensitivity. To investigate this, liposarcoma cell lines SW872 and SW982 were treated with either PI-103 (0.5 μmol) alone, chemotherapy drug (cisplatin or doxorubicin) alone, or the combination of chemotherapy drug and PI-103 for 48 hours. The data showed that the addition of PI-103 to these liposarcoma cell lines concurrently exposed to either cisplatin or doxorubicin resulted in a synergistic effect on cell growth inhibition in liposarcoma cells (Figure 5).

## Discussion

Identification of specific genetic changes in liposarcoma may help develop potential targeted therapy approaches using small-molecule inhibitors. We compared 37 liposarcoma or lipoma patient tumor tissue samples using a site-directed mutational profiling platform that has been implemented for clinical tumor genotyping. The characteristics of clinical and pathologic samples of liposaroma in this study are similar to those described in our previously published studies [3]. Among the 150 common mutations that were evaluated across 15 oncogene and tumor suppressor genes in the screening panel, the *PIK3CA* gene, which encodes the p110 alpha subunit of PI3K, was the only gene that was found to be mutated in liposarcoma tissues.

The PI3K pathway is widely dysregulated in human cancer and many studies have shown this pathway to be vital to the growth and survival of cancer cells [24], [25]. Multiple mechanisms have been found to induce PI3K signaling, such as activating mutation or amplification of key genes in this pathway (*PIK3CA* and *AKT1*) or loss of function of the negative modulator *PTEN* [28], [29]. PTEN down-regulation has been shown as a malignant signature in liposarcoma and response to PI3K pathway inhibition either with rapamycin alone or in combination with the multikinase inhibitor sorafenib [18]. PI3K can also be activated by upstream receptor tyrosine kinases (RTKs) [24], [25]. Aberrant AKT activation has also been shown in a zebrafish model of well-differentiated liposarcoma [5]. For these reasons, several small molecular inhibitors that target the PI3K/AKT signaling pathway have been tested using both *in vitro* and *in vivo* cancer models [24], [25].

In our study, activating mutations in the *PIK3CA* gene were found in 4 out of 18 human liposarcoma samples (22%) and in none of the 19 benign lipomas or the liposarcoma cell lines tested. These data are comparable with previously reports showing a *PIK3CA* mutation rate of 14–18% in myxoid/round-cell liposarcomas [17], [30]. The *PIK3CA* mutations in were also associated with AKT activation and poor clinical outcomes [30]. Interestingly, in our current study, AKT and mTOR activation were also seen in *PIK3CA* non-mutant liposarcoma samples. As PI3K signaling is activated in human cancers via several different mechanisms, these data suggest that alternative mechanisms other than *PIK3CA* mutation are also important for PI3K activation in liposarcoma. Further investigations are required to identify these alternative mechanisms, which could include PTEN or RTK status in these *PIK3CA* non-mutant liposarcomas. Taken together, these findings suggest that activation of the AKT/mTOR pathway in liposarcoma can occur either through activating *PIK3CA* gene mutations or by alternative cancer mechanisms.

We recognize the limitations of our clinical genotyping strategy where many other important mutations or tumor drivers are not detected [19]. The mutational profiling platform utilized was developed to simultaneously query across 150 site-specific mutations that are commonly mutated in cancer and have therapeutic implications. Therefore, alternative genetic mechanisms that are less frequent or have uncertain clinical benefit were not evaluated. These may include gene amplification, insertion, deletion or translocation (eg, FUS-CHOP or EWS-CHOP), as well as mutation in other cancer genes.

Western blot analysis confirmed the downstream activation of AKT in both *PIK3CA* mutant and non-mutant liposarcoma samples, supporting this pathway’s importance in liposarcoma tumorigenesis. Another new approach of our study is to inhibit PIK3 signaling with a dual inhibitor of PI3K/mTOR in liposarcoma. PI-103 effectively inhibited the activation of PI3K/AKT in liposarcoma cell lines and induced apoptosis. The magnitude of effects on survival was similar to that seen with treatment using chemotherapy. Importantly, when PI-103 and chemotherapy were combined, strong synergistic activity was noted. The results suggest PI-103 may lower the apoptotic threshold of chemotherapy drug in liposarcoma cells by targeting PIK/AKT. PI-103 has been used in preclinical studies and has demonstrated promising inhibitory results for various types of cancer [27], [31]–[33]. A more recent study also showed treatment of liposarcoma xenograft models with either the PI3K/AKT/mTOR pathway inhibitor rapamycin alone or in combination with the multi-kinase inhibitor sorafenib, all xenografts responded with increased lipid content and a more differentiated gene expression profile [34].

In conclusion, as the exact efficacy of chemotherapy in liposarcoma has not been clearly established, with the failure rate being high [1], [35], the results showed of this study suggest that chemotherapy drugs, when combined with PIK3 inhibitor PI-103 combination may prove an effective approach for the treatment of liposarcoma.
